# Infective Endocarditis in Ireland: A Nationwide Analysis

**DOI:** 10.3390/jcm15176525

**Published:** 2026-08-24

**Authors:** Aoife Maher, Eoghan de Barra, Fidelma Fitzpatrick, Fiona Boland, James O’Connell, Brendan McAdam

**Affiliations:** 1 Beaumont Hospital, D09 V2N0 Dublin, Ireland; 2 School of Postgraduate Studies, Royal College of Surgeons Ireland (RCSI), University of Medicine and Health Sciences, D02 YN77 Dublin, Ireland; 3 Department of International Health and Tropical Medicine, Royal College of Surgeons Ireland (RCSI), University of Medicine and Health Sciences, D02 YN77 Dublin, Ireland; 4 Department of Infectious Diseases, Beaumont Hospital, D09 V2N0 Dublin, Ireland; 5 Department of Clinical Microbiology, Royal College of Surgeons Ireland (RCSI), University of Medicine and Health Sciences, D02 YN77 Dublin, Ireland; 6 Department of Clinical Microbiology, Beaumont Hospital, D09 V2N0 Dublin, Ireland; 7 Data Science Centre, School of Population Health, Royal College of Surgeons Ireland (RCSI), University of Medicine and Health Sciences, D02 YN77 Dublin, Ireland; 8 School of Medicine, University of Galway, H91 TK33 Galway, Ireland

**Keywords:** infective endocarditis, mortality, cardiac surgery

## Abstract

**Introduction**: Infective endocarditis (IE) remains a serious life-threatening condition associated with substantial morbidity and mortality. Within the Republic of Ireland, there is limited national data regarding the clinical and economic impact of IE. This study aimed to describe IE in Ireland. **Methods**: We conducted a retrospective cohort study using national Hospital Inpatient Enquiry (HIPE) data accessed through the National Quality Assurance and Improvement System (NQAIS). All admissions with a principal diagnosis of acute or subacute infective endocarditis (ICD10: I33.0, I33.8) from 1 January 2018–30 July 2025 were included. Duplicate episodes, incomplete records, nonemergency admissions, and cases without documented transoesophageal echocardiography (TOE) were excluded. Demographic, clinical, procedural, and outcome variables were explored. Costs were assigned using Healthcare Pricing Office guidance for length of stay (LOS). **Results**: A total of 441 patients met inclusion criteria. The mean age was 64.1 years (SD 17.0), and 75.5% were male. Surgical intervention occurred in 18.4% of cases. Surgically managed patients were younger than medically managed patients (mean age 58.8 years vs. 65.7 years, *p* < 0.001) and experienced a longer LOS (mean of 51 days vs. 36 days, *p* < 0.001). The overall in-hospital mortality rate was 9.5% (n = 42). On multivariable analysis, higher comorbidity burden (measured by Charlson comorbidity index (CCI)) was independently associated with in-hospital mortality (OR 1.09, 95% CI 1.06–1.13, *p* < 0.001). The total expenditure was €20.6 million. **Discussion**: IE in Ireland affects predominantly older, comorbid patients and is associated with significant healthcare resource utilization. In-hospital mortality rates appear favourable compared with published international series.

## 1. Introduction

Infective endocarditis (IE) is a complex pathology associated with significant morbidity and mortality. The diagnosis and treatment of IE remain a considerable clinical challenge owing to its heterogeneous presentation, its complex microbiological profile, and the diversity of patients it affects in typically comorbid populations [1,2,3,4]. The incidence of IE appears to be rising globally, with an estimated incidence of 3–13.8 per 100,000 population per year, though this varies considerably across regions and healthcare settings [2,5,6,7,8].

The epidemiology of IE has undergone a marked transformation in recent decades. Those most at risk are now older and frailer and carry a greater burden of comorbid disease [9]. Intravenous drug use remains a risk factor for IE, with its occurrence varying depending on geographical location, with increasing prevalence in the United States [10]. Rheumatic heart disease secondary to streptococcal infection was historically the predominant predisposing condition. In high-income countries, however, the advent of antibiotics has led to a dramatic reduction in rheumatic disease, replaced by a rise in cases related to degenerative valvular disease, prosthetic heart valves (both surgical and percutaneous), and cardiac implantable electronic devices (CIEDs). This shift has been accompanied by a change in causative pathogens, most notably a rise in staphylococci, as well as the emergence of antimicrobial-resistant organisms, presenting ever-greater challenges in treatment [11]. By contrast, the epidemiological landscape of IE in low-income countries remains largely unchanged, continuing to be driven predominantly by rheumatic heart disease and streptococcal infection [3,12].

These demographic and microbiological changes have occurred contemporaneously with significant advances in clinical practice, including improvements in cardiac imaging and cardiac surgery, the establishment of dedicated multidisciplinary IE teams and the adoption of guideline-directed care [13,14]. Despite this, in-hospital mortality has remained relatively stable at 15–30% [15], likely reflecting the increasing complexity of cases and the greater frailty of affected patients [16]. Complications are common and clinically significant, encompassing cardio-embolic events with neurological sequelae, heart failure and perivalvular abscesses frequently requiring prolonged, intensive, and costly medical care. The average hospital length of stay (LOS) for IE is considerable, with a median ranging from 20 to 41, and a significant proportion of patients require cardiac surgery, further adding to the clinical and financial burden [17,18,19,20]. From a healthcare system perspective, IE represents a resource-intensive diagnosis requiring significant monitoring and treatment, with costs driven not only by the acute admission (which may include surgery) but also by the need for long-term intravenous antibiotic therapy, repeat echocardiography and radiological imaging, multidisciplinary follow-up, and the frequent need for rehabilitation [21,22].

In Ireland, the current estimated population is 5.3 million people [23]. There are four centres which perform adult cardiothoracic surgery: two in Dublin, one in Cork and one in Galway [24]. As the population ages and the prevalence of age-related predisposing conditions grows, the overall burden of IE on the healthcare system will likely increase. Despite this, there is a paucity of published data at a national level regarding the incidence, clinical profile, and outcomes of IE, and international data may not be representative of the Irish context. This represents a significant gap in the evidence base to guide national healthcare planning and resource allocation. The present study aimed to address this by providing a contemporaneous national-level analysis of the epidemiology of IE in Ireland.

### Aims

The aim of this study was to characterise the epidemiology of IE in Ireland by examining rates of surgical intervention, inpatient mortality, and length of stay with associated costs.

## 2. Methods

### 2.1. Study Design

This was a retrospective cohort study using data from the Hospital Inpatient Enquiry (HIPE) system, a national database containing basic demographic, administrative and clinical data on all inpatient discharges from publicly funded hospitals in the Republic of Ireland. The National Quality Assurance and Improvement System (NQAIS) provides online analytical access to HIPE data. Diagnoses are coded using the International Classification of Disease 10th Revision (ICD-10), and procedures are coded using the Australian Coding of Health Interventions (ACHI). Access to NQAIS was obtained using the standardised Health Service Executive [25] protocol. No further ethical approval was required.

### 2.2. Data Criteria

All episodes of care related to IE from 1 January 2018 to 30 July 2025 were identified in the public hospital system only. This period was chosen because at the time of data extraction, the 1st of January 2018 was as far back as the database went and the 30th of July was the most recent date with up-to-date available data. An episode was included when acute or subacute IE (ICD-10 I33.0 and I33.8 codes identified via Centre for Disease Prevention [26,27]) was recorded as the principal diagnosis. Patients’ unique NQAIS number, age, sex, episode of care type, admitting hospital, secondary diagnoses, procedures performed, LOS, Charlson Comorbidity Index (CCI), diagnosis-related grouping (DRG), specialty and discharge destination were extracted. Episodes of care were classified as elective (scheduled admission), same day (unscheduled admission discharged on the day of arrival), or emergency (unscheduled admission requiring inpatient stay). DRG codes are a system used to classify inpatient and day-case treatments and to calculate payments under Ireland’s public health system. Each DRG code corresponds to a specific treatment and the cost associated with it. This code determines the payment amount for the treatment that the hospital will receive. Given the number of interhospital transfers, patient episodes were matched—and identified as a single case—unless a patient’s episode from admission to discharge was fully accounted for they were excluded from this study. The CCI is a method of quantifying morbidity and predicting mortality by classifying or weighting the presence of between 0 and 17 comorbid conditions. Patients with an index of 0 are said to have none of the comorbidities contained in the CCI, while, for example, patients with moderate chronic kidney disease would have an index of 2. These values are not age adjusted in our study. The CCI data was derived directly from the NQAIS database. European Society of Cardiology (ESC) guidelines were used to identify and classify [28] risk factors as cardiac or non-cardiac. Costs were assigned using 2024 Healthcare Pricing Office guidance based on DRG, admission type, length of stay (mean LOS) and episode complexity [29]. Cases were excluded if transoesophageal echocardiogram (TOE) was not recorded; this was to ensure that only cases of IE were included in the analysis, as many cases coded as endocarditis, had procedures completed, including lumbar puncture, which are linked to a different speciality. The NQAIS database does not include details on patients who underwent a transthoracic echocardiogram. Only emergency admissions were selected given the number of elective cases with a length of stay of 0.5 days, which did not reflect an IE admission. A case was considered a readmission if readmitted within the NQAIS coding definition of readmission. All included readmissions were <30 days.

### 2.3. Statistical Analysis

Data was managed in Microsoft Excel. ACHI codes were used to identify TOE procedures. Risk factors, causative pathogens and surgical interventions were identified using the secondary diagnosis and procedures listings. Statistical analysis was performed using Stata v19 (StataCorp. 2025. Stata Statistical Software: release 19. College Station, TX, USA: StataCorp LL). Continuous variables are reported as mean ± standard deviation (SD) or median with interquartile range (IQR) depending on distribution. Categorical variables are reported as frequencies and percentages. Group comparisons were performed using the Mann–Whitney U test for non-normally distributed continuous variables and *t* tests for normally distributed continuous variables. Pearson’s chi-squared test or Fisher’s exact test were used to assess associations between categorical variables. An analysis of variance was used to compare subgroups of patients with and without risk factors and pathogens. A two-tailed *p*-value < 0.05 was considered statistically significant.

## 3. Results

### 3.1. Description of Episodes of Care

Between 1 January 2018 and 30 July 2025, there were 1706 episodes of care in which IE was recorded as the principal diagnosis. After matching related episodes, there were 1239 individual cases. The exclusion of cases with incomplete documentation reduced the dataset to 833, with a further reduction to 768 when the cohort was limited to emergency admissions and further reduction to 441 with a TOE documented. There were 16 elective cases excluded who had a TOE; of these, only 1 underwent possible surgery linked to IE. The number of cases varied across the study period from 57 in 2018 to 62 in 2024, with 21 in 2025 (incomplete year of data). The lowest was 47 cases in 2021 and the highest 70 in 2019.

The mean age of patients was 64.1 years (SD 17.0, range 18–97). The female-to-male ratio was 108:333 (24.5%:75.5%). Most patients (97.9%) were admitted from home, with the remaining admissions from temporary accommodation, nursing homes, or convalescent care. A total of 289 (65.5%) were admitted to a level 4 hospital and 152 (34.5%) to a level 3 hospital. Level 3 hospitals provide both elective services and admit undifferentiated medical and surgical patients, whilst level 4 hospitals admit undifferentiated medical and surgical patients and take referrals from other hospitals [30]. They provide complex specialist care and have a category 3 or 3S on-site ICU. Transfer between hospitals occurred in 70 (15.8%) patients. Although NQAIS does not capture transfer indications, the pattern suggests possible referral for cardiothoracic surgery, as 59 (84.3%) involved transfer to a cardiothoracic centre. Readmissions due to complications accounted for 22 (5%) patients.

Risk factors were documented in 257 cases. Following European Society of Cardiology (ESC) guidelines [28], risk factors were classed as cardiac (43.5%), non-cardiac (11.3%), or both (3.4%). A more detailed breakdown is provided in Table 1.

Causative pathogens were documented in 24.7% (n = 109) of cases; *Staphylococcus aureus* accounted for 50.5% (n = 55) of these, followed by a grouping on NQAIS of *Streptococci* species and *Enterococci* species at 19.27% (n = 21). Separately, *Streptococci* species alone accounted for 17.43% (n = 19), *Enterococci* species alone for 5.50% (n = 6) and others for 7.35% (n = 8 (n = 2 other, n = 6 Gram-negative species)).

### 3.2. Surgical Intervention

The overall surgical rate was 18.4% (n = 81). The aortic valve (AV) was the most frequently treated site, with AV replacement (AVR) accounting for 40% (n = 32) of all surgical procedures. A further 30% (n = 24) involved combined AVR with mitral valve repair or replacement or AVR with coronary artery bypass grafting. CIED removal comprised 10% (n = 8) of interventions.

Patients who underwent surgery were significantly younger than those managed medically (57 ± 16 vs. 66 ± 17 years, *p* < 0.001). There were no statistically significant differences in sex distribution, inpatient mortality, or CCI between groups (Table 2).

In a sensitivity analysis excluding participants with a LOS greater than 100 days, 347 cases remained in the no-surgery group and 75 in the surgery group. The results were consistent with the primary analysis, and there was no evidence of a change in interpretation (Supplementary Table S1).

### 3.3. Mortality Analysis

The overall in-hospital mortality was 9.5% (n = 42). Most patients (81.4%) were discharged home. Other discharge destinations included a nursing home (6.8%), self-discharge (1.6%) or hospice (0.7%). Of the patients who died, 19.05% of patients died within two weeks of admission. A total of 50% of patients who died had died within a month, whilst 47.12% of survivors had been discharged home at the same time point. A univariate analysis was conducted to evaluate several variables for an association with inpatient mortality. Only the CCI demonstrated a significant difference (Table 3). Patients who died had higher comorbidity scores. On multivariable logistic regression analysis, a higher CCI remained independently associated with an increased odds of inpatient mortality (OR: 1.09, 95%CI: 1.06—1.13; *p* < 0.001).

In sensitivity analyses excluding admissions with LOS > 100 days, the estimated effects of age, sex, comorbidity burden and surgery were materially unchanged (Supplementary Table S2).

### 3.4. Length of Stay Analysis and Costing

The overall mean LOS was 38.5 ± 29.5 days. Across the study period, the annual mean LOS varied from 36.9 days in 2020 to 43.9 days in 2024. There was no evidence of a statistically significant difference in LOS between patients who died during admission and those who survived (OR:0.99, 95%CI: 0.96–1.01, Table 3). However, LOS was significantly longer in patients who underwent surgical intervention compared with those managed medically (51 ± 35 days versus 36 ± 27 days, *p* < 0.001). ICU length of stay was significantly longer in surgical patients (median 6 days, IQR 2–14, n = 81) compared with non-surgical patients (median 0 days, IQR 0–3, n = 360); *p* < 0.001.

Costing was available for 416 cases of IE. Cases impacted by DRG coding changes from 2024 onward were excluded, as were episodes whose DRGs were not listed in the HPO guidelines and therefore had no available costing. The total cost of IE was €20,568,656, amounting to an average of €49,444 per case of endocarditis, based on LOS. The average cost for those who underwent surgery was higher, at €69,406, whilst those who did not undergo surgery had an average cost of €45,543.

### 3.5. Subgroup Analysis A: Comparison of Patients with and Without Identifiable Risk Factors and/or Pathogens

We compared four subgroups based on the presence or absence of clinical risk factors and microbiologically identified pathogens. These comprised patients with both a risk factor and a pathogen (n = 62), patients with a pathogen but no identified risk factor (n = 47), patients with a risk factor but no pathogen isolated (n = 195) and patients with neither a risk factor nor pathogen documented (n = 137). Descriptive statistics for these groups are summarised in Table 4.

### 3.6. Subgroup Analysis B: Patients Not Included in Primary Analysis

The highest inpatient mortality was observed among patients with a pathogen but no identified risk factor (17%), while the lowest mortality occurred in patients with neither a risk factor nor a pathogen identified (7%). However, there was no statistically significant evidence of a difference in mortality across the four subgroups. There was also no statistically significant difference in mortality across pathogen subgroups; whilst *S. aureus* accounted for 21.6% of deaths with identifiable pathogens, it did not reach significance (*p* = 0.16).

CCI scores were highest among female patients from the subgroup who had a pathogen isolated but no identified risk factor, although the overall comparison of CCI across all subgroups was not statistically significant, and the interaction between sex and subgroup was also non-significant (*p* = 0.11). Rates of surgical intervention differed significantly between subgroups (χ^2^(3) = 9.24, *p* = 0.03). Patients with both a risk factor and a pathogen identified were most likely to undergo surgery (24%), whereas those with a pathogen but no identified risk factor had the lowest intervention rate (9%). Prior to adjustment for multiple testing, three pairwise comparisons were statistically significant (risk factor + pathogen vs. pathogen only, *p* = 0.033; pathogen only vs. risk factor only, *p* = 0.030; risk factor only vs. neither risk factor nor pathogen, *p* = 0.030). After applying a Bonferroni correction, none of the pairwise differences remained statistically significant.

Overall, patients with a pathogen isolated but no identified risk factor were less likely to undergo surgical intervention, had a higher comorbidity burden (particularly among females), and experienced higher inpatient mortality than the other subgroups.

In comparison, there were 327 emergency admissions without TOE completed, and the average age was 66.4 years. The mortality rate was 16.37%, and 2.75% underwent cardiac surgery. The average CCI was 6.5. Table 5 compares the primary group of patients with TOE with those not included due to not undergoing a TOE.

Of those who died and who had a primary diagnosis of IE, did not undergo a TOE but did undergo a separate procedure, endoscopy was the most common (12.72%), followed by biopsy of the lung (3.64%); other procedures included lumbar puncture, bronchoscopy, embolectomy and aortic valve replacement. The patients who died were older, with a mean age of 79.05, with a higher CCI (mean 12.7). The elective cases with TOE accounted for only 16 cases, 3 of which underwent a cardiac procedure.

## 4. Discussion

This national-level analysis provides an up-to-date overview of IE in Ireland, highlighting a predominantly older, male cohort with a relatively low rate of surgical intervention and an in-hospital mortality of 9.5%. Comorbidity burden emerged as the primary independent predictor of mortality, underscoring the clinical severity of this population and the substantial healthcare resources required, including prolonged hospital stays and significant associated costs, whilst younger age emerged as the predictor of undergoing surgery. These findings contribute important epidemiological and health service insights within an Irish context and allow for meaningful comparison with the existing international literature. Furthermore, subgroup patterns—particularly higher mortality among patients without identifiable risk factors but with a documented pathogen—warrant further investigation.

The demographic characteristics of this cohort align with the contemporary epidemiological shift observed across high-income countries, in which IE increasingly occurs among older, predominantly male individuals with a significant burden of comorbid disease. The mean age of 64.1 years and a male-to-female ratio of approximately 3:1 mirror findings from major European datasets [4]. The pronounced male preponderance has been attributed in part to higher rates of predisposing conditions among men, such as degenerative valvular disease, CIED implantation, and injecting drug use [31,32,33,34,35]. The finding that 97.9% of patients were admitted from home, rather than from healthcare facilities, is noteworthy and may suggest under-coding of healthcare-associated risk factors, such as prior hospitalisation, haemodialysis, or central venous access, an acknowledged limitation inherent to analysis relying on administrative databases.

The risk factor profile observed in this cohort mirrors the broader epidemiological transition described internationally. Valvular heart disease, prosthetic valve endocarditis, and CIED-related endocarditis collectively accounted for most identifiable cardiac risk factors, reflecting the ageing population and the expanding use of cardiac prostheses and implantable devices in modern cardiology practice [35,36,37], a trend that poses growing challenges. Intravenous drug use (IVDU) accounted for 3.9% of cases in this cohort; this figure is likely an underestimate given the recognised difficulties in accurately coding substance misuse in administrative datasets. This is a relevant consideration, as IVDU-related IE typically exhibits distinct features, including a predilection for right-sided valvular involvement, younger patient age, and recurrent infection risk, all of which carry significant challenging implications for surgical decision-making [10,18,38]. Among cases in which a causative pathogen was identified, the microbiological profile was dominated by *S. aureus* (50.5%), followed by streptococcal species and enterococcal species. This distribution is consistent with the global epidemiological shift from streptococcal to staphylococcal predominance in IE, a trend attributable to the decline in rheumatic heart disease, the increase in healthcare-associated IE, and the expanding use of CIED and prosthetic valves [2,39,40,41]. *S. aureus* is well recognised as the most virulent IE pathogen, associated with higher incidence of embolic complications, perivalvular extension, and mortality compared with other pathogens [39,42,43,44,45,46]. However, causative pathogens were only documented in 24.7% of cases, likely reflecting prior antibiotic therapy before blood culture collection and variability in culture techniques. It also suggests that coding may significantly underestimate pathogen identification rates.

The overall surgical intervention rate of 18.4% observed in this cohort is lower than the 25–40% typically reported for management of IE during index admission [4,47,48,49]. This discrepancy may reflect under-ascertainment due to reliance on ACHI procedure coding. The administrative nature of NQAIS also limits insight into clinical variables such as echocardiographic findings, haemodynamic status, or heart failure severity, all of which underpin surgical decision-making in current ESC guidelines [28]. Additionally, some patients may have been referred for surgery but were unable to undergo intervention due to clinical deterioration, death, or frailty in the context of significant comorbidity. As expected, surgical patients were significantly younger and experienced longer inpatient stays, reflecting the complexity of postoperative recovery, prolonged antibiotic therapy, and intensive monitoring requirements. Reassuringly, there was no evidence of a difference in inpatient mortality between surgical and medical management groups, suggesting appropriate selection of operative candidates and alignment with international experience.

The overall inpatient mortality of 9.5% is lower than the 10–30% reported across the European literature, although comparisons must be interpreted with caution given methodological differences between coding data and prospective clinical registries [4,8,9,50,51,52,53,54,55]. A nationwide US database reported a similar inpatient mortality of 9.8%, suggesting comparable outcomes in large administrative databases [17]. The lower observed mortality may, in part, reflect a survivor bias inherent to administrative data, whereby patients with the most severe presentations may die before coding is completed or may be excluded due to incomplete documentation. Comorbidity burden, as quantified by the CCI, was the sole independent predictor of inpatient mortality, with each unit increase associated with an 11% increase in the odds of death, findings consistent with growing evidence highlighting comorbidity as a key determinant of outcomes in IE [53,54,56]. The absence of an independent association between age and mortality after adjusting for comorbidity highlights the importance of undertaking individualised, holistic patient assessment rather than relying on age-based prognostication. Notably, neither surgical intervention nor LOS demonstrated an independent association with mortality in this cohort.

The median LOS of 32 days (mean 38.5 days, 36.9 to 43.9) underscores the considerable and prolonged resource burden of IE. Reported LOS in other administrative and registry analyses varies widely, from 7 to 35 days [33,53,57,58,59,60], a range likely influenced by differences in healthcare systems, availability of outpatient parenteral antibiotics, and the adoption of oral step-down regimens; which explains why LOS is may be shorter than expected for those patients on outpatient treatment. European studies generally report median stays of 18–35 days, more consistent with Irish figures, whereas shorter LOS is often observed in studies from the US [17,60]. As expected, LOS was considerably longer among surgical patients (mean 51.2 days versus 35.6 days), reflecting the complexity of postoperative recovery. However, published evidence is mixed regarding whether surgical intervention consistently results in longer LOS [47,61], limiting the extent to which firm conclusions can be drawn from LOS comparisons alone.

From a health economics perspective, the total expenditure of €20,568,656 across 416 costed cases, equating to a mean cost of €49,444 per admission and an average daily cost of €1563, highlights the substantial financial burden that IE places on the Irish healthcare system. The average cost per day was €1563. US data from 2016 reported comparable costs, with a median LOS of 10 days associated with expenses of €37,664 [17]. Comparable per-admission costs typically range from €20,000 to €50,000, influenced by the need for surgical management [18,21,62,63,64], though contemporary cost analyses remain limited, with few publications after 2018. As the population ages and predisposing conditions become increasingly prevalent, the aggregate cost burden of IE is expected to rise, with a need for additional rehabilitation in frailer adults, reinforcing the importance of targeted prevention strategies, timely diagnosis, and optimised antimicrobial therapy. Emerging evidence supporting earlier oral antibiotic switch in clinically stable patients not requiring surgery suggests a potential way of reducing LOS and cost [20,57].

The subgroup analysis stratifying patients by identifiable risk factors and documented pathogens revealed several clinically relevant patterns. Patients in whom a pathogen was identified without an associated risk factor demonstrated the highest inpatient mortality (17%) and comorbidity burden while being least likely to undergo surgical intervention (9%). This subgroup may represent diagnostically challenging cases in whom IE occurs without recognised predisposing structural cardiac disease, potentially reflecting undocumented healthcare-associated exposure, cryptogenic bacteraemia, or incomplete risk factor ascertainment inherent to administrative datasets. The lower surgical intervention rate in this high-mortality group warrants further investigation and may reflect clinical decision-making influenced by high operative risk due to comorbidity.

Conversely, patients with both an identified risk factor and an identified pathogen were most likely to undergo surgical intervention (24%), consistent with the expectation that a clearly defined microbiological and structural diagnosis facilitates more definitive surgical planning. Although the difference in surgical rates across subgroups was attenuated after Bonferroni correction, the pattern represents a hypothesis-generating finding warranting prospective validation.

This study has several limitations inherent to its retrospective design and reliance on administrative data. The HIPE system captures diagnostic and procedural codes rather than clinical variables, precluding detailed analysis of echocardiographic findings, microbiological susceptibility profiles, antibiotic regimens, complications such as embolic events or heart failure class, or adherence to ESC guideline-based management. The low rate of documented pathogens (24.7%) likely reflects coding limitations rather than true microbiological yield, and similarly, risk factor ascertainment may be incomplete. The NQAIS database contains data from patient discharge letters, and given that interns largely carry out discharge documentation, the inclusion of documentation of causative pathogens and risk factors may not have always been completed. The requirement for documented TOE as an inclusion criterion, while intended to strengthen diagnostic specificity, may have introduced selection bias by excluding patients managed in centres with limited TOE availability or those too unstable to undergo the procedure. The exclusion of non-emergency and incomplete episodes limits the generalisability of findings. Not all cases could be costed, which may have led to over- or underestimation of the cost of care, especially given the current economic climate. The exclusion of elective cases (n = 16) and those patients without a TOE is a further limitation. Emergency patients whose primary diagnosis was endocarditis but who did not have a TOE (n = 327) underwent other procedures: 42.8% (140) engaged with occupational therapy and physiotherapy (NQAIS considers this a procedure in coding), 38.53% (126) had no procedure of any description, 5.8% had endoscopy, 3.7% had biopsy of liver/skin/lung, and 9.2% had other procedures, including lumbar puncture, dialysis, red cell transfusion, drug detoxification and management of non-invasive ventilation. Given the assortment of procedures, it is challenging to claim that these are cases of endocarditis. Patients without a TOE who died were significantly older and more comorbid than those who died with a TOE. However, given the lack of detail on these patients, it is very difficult to discern whether these patients had IE or were incorrectly coded from a diagnosis viewpoint. Nonetheless, the scale and national coverage of the dataset, including nearly eight years and encompassing all publicly funded hospitals in Ireland, provide a uniquely comprehensive national perspective on the burden and outcomes of IE at a population level.

## 5. Conclusions

In conclusion, this national analysis provides the most comprehensive contemporary overview of IE in Ireland to date, highlighting its substantial clinical complexity, prolonged hospitalisation, and significant economic burden. The findings highlight the central role of comorbidity in shaping outcomes and reveal important variation in surgical intervention and microbiological documentation that warrants further prospective investigation. As the population ages and the prevalence of predisposing cardiac and healthcare-associated factors continues to rise, the burden of IE is likely to increase, reinforcing the need for strengthened prevention strategies, improved diagnostic pathways, and optimised antimicrobial stewardship. Investment in high-quality national data collection and prospective research will be essential to guide future policy, enhance patient outcomes, and support more efficient use of healthcare resources, given the significant limitations outlined in our study and the lack of a national IE registry in Ireland thus far.

## Figures and Tables

**Table 1:** Identified risk factors for infective endocarditis in Ireland, 1 January 2018 and 30 July 2025.

Risk Factor	N (%)
Valvular disease	88 (19.9%)
Cardiac implantable electronic devices (CIEDs)	46 (10.4%)
Prosthetic valve	46 (10.4%)
Persons who inject drugs	16 (3.9%)
Prosthetic valve and CIEDs	12 (2.7%)
Congenital heart disease	10 (2.3%)
Immunocompromised	9 (2.0%)
Valvular disease and non-cardiac risk factors	9 (2.0%)
Dental disease	8 (1.8%)
Dialysis/long line	7 (1.6%)
CIED and other risk factors	3 (0.7%)
Prosthetic valve and non-cardiac risk factors	3 (0.7%)

**Table 2:** Multivariable logistic regression findings in cases of infective endocarditis stratified by surgical treatment.

Variable	Surgery: Yes (N = 81)	Surgery: No (N = 360)	*p*-Value *	Adjusted OR (95% CI)	*p*-Value
**Sex, n (%)**			0.27		
Female	16 (20%)	94 (26%)	—	Reference	—
Male	65 (80%)	266 (74%)		1.48 (0.80–2.74)	0.207
**Age in years, mean (SD)**	58.8 (15.8)	65.7 (16.9)	<0.001	0.97 (0.95–0.98)	<0.001
**Charlson Comorbidity Index, mean (SD)**	8.5 (8.8)	7.3 (9.3)	0.30	1.03 (0.99–1.05)	0.063

* χ^2^ tests or *t* tests as appropriate.

**Table 3:** Characteristics of patients with infective endocarditis by inpatient mortality and multivariable logistic regression analysis.

Variable	Mortality: Yes (N = 42)	Mortality: No (N = 399)	*p*-Value *	Adjusted OR (95% CI)	*p*-Value
**Sex, n (%)**			0.91		
Female	10 (24%)	100 (25%)	—	Reference	—
Male	32 (76%)	299 (75%)		1.23 (0.56–2.07)	0.609
**Mean age in years, (SD)**	66.9 (15.2)	63.8 (17.3)	0.26	1.00 (0.98–1.03)	0.687
**Charlson Comorbidity Index, mean (SD)**	15.8 (10.7)	6.7 (8.6)	<0.01	1.09 (1.06–1.13)	<0.001
**Surgery, n (%)**					
Yes	8 (19%)	73 (18%)		Reference	
No	34 (81%)	326 (82%)		1.03 (0.43–2.46)	0.951

* χ^2^ tests or *t* tests as appropriate.

**Table 4:** Characteristics of patients with infective endocarditis stratified by (1) risk factors (RF) present and pathogen identified, (2) no risk factor but pathogen identified, (3) risk factors present but no pathogen identified, and (4) neither risk factor nor pathogen identified.

		Total (Primary Group) n = 441	With RF and with Pathogen n= 62	No RF and with Pathogen n = 47	With RF and No Pathogen n= 195	No RF and No Pathogen n = 137	*p*-Value *
**Sex, n (%)**	Male	333 (75%)	45 (73%)	37 (81%)	145 (74%)	105 (77%)	0.74
	Female	108 (25%)	17 (27%)	9 (19%)	50 (26%)	32 (23%)	
**Mean age in years, (SD)**		64.1 (17.1)	64.7 (17.3)	67.7 (15.4)	62.3 (17.5)	65.0 (16.8)	0.20
**Inpatient mortality, n (%)**		42 (10%)	9 (15%)	8 (17%)	15 (8%)	10 (7%)	0.09
**Surgical intervention n (%)**		81 (18%)	15 (24%)	4 (9%)	44 (23%)	18 (13%)	0.03
**Mean Charlson Comorbidity Index, (SD)**		7.5 (9.2)	9.5 (10.4)	8.9 (9.8)	7.7 (9.2)	6.0 (8.4)	0.05
**Mean Charlson Comorbidity Index, (SD)**	Male	7.3 (8.9)	8.8 (10.0)	7.3 (8.2)	7.9 (9.3)	5.8 (8.2)	0.11
	Female	8.3 (10.1)	11.4 (11.6)	15.4 (13.5)	7.2 (9.0)	6.5 (9.1)	
**Mean length of stay (days), (SD)**		38.5 (29.5)	41.4 (23.1)	37. 5 (24.3)	41.3 (29.3)	33.7 (33.4)	0.11

* χ^2^ tests or ANOVA as appropriate.

**Table 5:** Comparison of those with and without a TOE.

	With TOE (n = 441)	Without TOE (n = 327)
**Age, mean**	64.1	66.4 years
**Inpatient mortality**	42 (9.5%)	55 (16.81%)
**Cardiac surgery**	81 (18.4%)	9 (2.75%)
**Length of stay, mean (SD)**	38.5 days (29.5)	30.1 days (31.8)

## Data Availability

Data is not available publicly and may be requested from NQAIS directly.

## Supplementary Materials

The following supporting information can be downloaded at: https://www.mdpi.com/article/10.3390/jcm15176525/s1, Table S1: Characteristics of participants by surgical intervention and multivariable logistic regression analysis—Sensitivity analysis excluding length of stay >100 days; Table S2: Characteristics of participants by inpatient mortality and multivariable logistic regression analysis—Sensitivity analysis excluding length of stay >100 days.

## Abbreviations

ACHI	Australian Coding of Health Interventions
AV	Aortic valve
AVR	Aortic valve replacement
CCI	Charlson Comorbidity Index
CIED	Cardiac implantable electronic devices
DRG	Diagnosis-related group
ESC	European Society of Cardiology
HIPE	Hospital Inpatient Enquiry
ICD10	International Classification of Disease 10th Revision
IE	Infective endocarditis
IQR	Interquartile range
IVDU	Intravenous drug use
LOS	Length of stay
NQAIS	National Quality Assurance and Improvement System
OR	Odds ratio
RF	Risk factor
SD	Standard deviation
TOE	Transoesophageal echocardiography

## References

[1] Moreillon P., Que Y.A. (2004). Infective endocarditis. Lancet.

[2] Ambrosioni J., Hernández-Meneses M., Durante-Mangoni E., Tattevin P., Olaison L., Freiberger T., Hurley J., Hannan M.M., Chu V., Hoen B. (2023). Epidemiological Changes and Improvement in Outcomes of Infective Endocarditis in Europe in the Twenty-First Century: An International Collaboration on Endocarditis (ICE) Prospective Cohort Study (2000–2012). Infect. Dis. Ther..

[3] Ambrosioni J., Hernandez-Meneses M., Téllez A., Pericàs J., Falces C., Tolosana J.M., Vidal B., Almela M., Quintana E., The Hospital Clinic Infective Endocarditis Investigators (2017). The Changing Epidemiology of Infective Endocarditis in the Twenty-First Century. Curr. Infect. Dis. Rep..

[4] Habib G., Erba P.A., Iung B., Donal E., Cosyns B., Laroche C., Popescu B.A., Prendergast B., Tornos P., Sadeghpour A. (2019). Clinical presentation, aetiology and outcome of infective endocarditis. Results of the ESC-EORP EURO-ENDO (European infective endocarditis) registry: A prospective cohort study. Eur. Heart J..

[5] Momtazmanesh S., Saeedi Moghaddam S., Malakan Rad E., Azadnajafabad S., Ebrahimi N., Mohammadi E., Rouhifard M., Rezaei N., Masinaei M., Rezaei N. (2021). Global, regional, and national burden and quality of care index of endocarditis: The global burden of disease study 1990–2019. Eur. J. Prev. Cardiol..

[6] Chen H., Zhan Y., Zhang K., Gao Y., Chen L., Zhan J., Chen Z., Zeng Z. (2022). The Global, Regional, and National Burden and Trends of Infective Endocarditis From 1990 to 2019: Results From the Global Burden of Disease Study 2019. Front. Med..

[7] Martiny S.K., Schmidt M., Povlsen J.A., Søgaard K.K., Bøtker H.E., Sørensen H.T. (2025). Incidence rate of infective endocarditis by socioeconomic position: A Danish nationwide cohort study (2000–2022). Lancet Reg. Health—Eur..

[8] Cahill T.J., Prendergast B.D. (2016). Infective endocarditis. Lancet.

[9] Murdoch D.R., Corey G.R., Hoen B., Miró J.M., Fowler V.G., Bayer A.S., Karchmer A.W., Olaison L., Pappas P.A., Moreillon P. (2009). Clinical presentation, etiology, and outcome of infective endocarditis in the 21st century: The International Collaboration on Endocarditis-Prospective Cohort Study. Arch. Intern. Med..

[10] Mori M., Brown K.J., Bin Mahmood S.U., Geirsson A., Mangi A.A. (2020). Trends in Infective Endocarditis Hospitalizations, Characteristics, and Valve Operations in Patients With Opioid Use Disorders in the United States: 2005–2014. J. Am. Heart Assoc..

[11] Wang A., Gaca J.G., Chu V.H. (2018). Management Considerations in Infective Endocarditis: A Review. JAMA.

[12] Noubiap J.J., Nkeck J.R., Kwondom B.S., Nyaga U.F. (2022). Epidemiology of infective endocarditis in Africa: A systematic review and meta-analysis. Lancet Glob. Health.

[13] Habib G., Lancellotti P., Antunes M.J., Bongiorni M.G., Casalta J.-P., Del Zotti F., Dulgheru R., El Khoury G., Erba P.A., Iung B. (2015). 2015 ESC Guidelines for the management of infective endocarditis: The Task Force for the Management of Infective Endocarditis of the European Society of Cardiology (ESC)Endorsed by: European Association for Cardio-Thoracic Surgery (EACTS), the European Association of Nuclear Medicine (EANM). Eur. Heart J..

[14] Tan C., Hansen M., Cohen G., Boyle K., Daneman N., Adhikari N.K.J. (2016). Accuracy of administrative data for identification of patients with infective endocarditis. Int. J. Cardiol..

[15] Leone S., Ravasio V., Durante-Mangoni E., Crapis M., Carosi G., Scotton P.G., Barzaghi N., Falcone M., Chinello P., Pasticci M.B. (2012). Epidemiology, characteristics, and outcome of infective endocarditis in Italy: The Italian Study on Endocarditis. Infection.

[16] Delle Karth G., Koreny M., Binder T., Knapp S., Zauner C., Valentin A., Honninger R., Heinz G., Siostrzonek P. (2002). Complicated infective endocarditis necessitating ICU admission: Clinical course and prognosis. Crit. Care.

[17] Alkhouli M., Alqahtani F., Alhajji M., Berzingi C.O., Sohail M.R. (2020). Clinical and Economic Burden of Hospitalizations for Infective Endocarditis in the United States. Mayo Clin. Proc..

[18] Fleischauer A.T., Ruhl L., Rhea S., Barnes E. (2017). Hospitalizations for Endocarditis and Associated Health Care Costs Among Persons with Diagnosed Drug Dependence—North Carolina, 2010–2015. MMWR Morb. Mortal. Wkly. Rep..

[19] Lone A.N., Khan M., Khan M.U., Khan S.U., Balla S. (2020). Abstract 28: Health Care Cost Burden of Infective Endocarditis in United States: A Nationwide Study. Circ. Cardiovasc. Qual. Outcomes.

[20] Ostergaard L., Pries-Heje M.P.H., Voldstedlund M.A.V., Bruun N.E.B., Povlsen J.A.P., Valeur Kober N.V.K., I Ihlemann N., Tuxen C.T., Hasselbalch R.H., Stahl A.S. (2024). Changes in length of hospital stay for infective endocarditis—Data from before and after the POET trial. Eur. Heart J..

[21] Bhandari R., Abdulhay N., Wiener R.C., Smith D., Fisher M. (2024). The rising cost of infective endocarditis in West Virginia. Epidemiol. Infect..

[22] Zaman J., Amritphale A., Malozzi C., Amritphale N., Sehgal M., Bassam O. (2021). Costs and predictors of early readmissions in patients with Infective Endocarditis. Utilizing the Nationwide Readmission Database. Biomed. Res. Clin. Rev..

[23] Central Statistics Office (2025). Population and Migration Estimation 2025. https://www.cso.ie/en/releasesandpublications/ep/p-pme/populationandmigrationestimatesapril2025/.

[24] Royal College of Surgeons Ireland (2026). Cardiothoracic Surgery Overview. https://www.rcsi.com/surgery/training/surgery/cardiothoracic-surgery/overview.

[25] HSE Guide to Using NQAIS Clinical for Research 2025. https://www.bing.com/ck/a?!&&p=13a127890926f0548fa6e97f33b63d25fe1115d93e9af5d40ddf9c088e069307JmltdHM9MTc3MzcwNTYwMA&ptn=3&ver=2&hsh=4&fclid=10affe86-e69b-6a88-1bf7-e8eee7ec6b98&psq=hse+nqais+explained&u=a1aHR0cHM6Ly93d3cucmNzaS5jb20vZHVibGluLy0vbWVkaWEvZmVhdHVyZS9tZWRpYS9kb3dubG9hZC1kb2N1bWVudC9zdXJnZXJ5L3ByYWN0aWNlL25jcC9zdXJnZXJ5L25xYWlzLWNsaW5pY2FsL2d1aWRlLXRvLXVzaW5nLW5xYWlzLWNsaW5pY2FsLWZvci1yZXNlYXJjaC5wZGY.

[26] Authority IHaACP ICD-10-AM/ACHI/ACS Thirteenth Edition 2026. https://www.ihacpa.gov.au/resources?search=ICD-10-AM/ACHI/ACS%20edition&type%5B10%5D=10&year%5Bmin%5D=&year%5Bmax%5D=&topics%5B31%5D=31&sort_by=relevance.

[27] Prevention CfDCa ICD-10-CM FY26, 1 October 2025. National Centre for Health Statistics—Centre for Disease Control and Prevention. https://ftp.cdc.gov/pub/Health_Statistics/NCHS/Publications/ICD10CM/2026/.

[28] Delgado V., Ajmone Marsan N., de Waha S., Bonaros N., Brida M., Burri H., Caselli S., Doenst T., Ederhy S., Erba P.A. (2023). 2023 ESC Guidelines for the management of endocarditis: Developed by the task force on the management of endocarditis of the European Society of Cardiology (ESC) Endorsed by the European Association for Cardio-Thoracic Surgery (EACTS) and the European Association of Nuclear Medicine (EANM). Eur. Heart J..

[29] ABF 2019 Admitted Patient Price List—Summary [Press Release]; Ireland: HSW2019. https://assets.hse.ie/media/documents/2019-admitted-patient-price-list-summary-daycase_plRNppu.pdf.

[30] HSE NDTaPa Medical Careers: About Ireland 2026. https://www.medicalcareers.ie/about/ireland/.

[31] Iung B. (2019). Endocardite infectieuse. Épidémiologie Physiopathol. Et. Anatomopathologie. La Presse Médicale.

[32] Toyoda N., Chikwe J., Itagaki S., Gelijns A.C., Adams D.H., Egorova N.N. (2017). Trends in Infective Endocarditis in California and New York State, 1998–2013. JAMA.

[33] Fedeli U., Schievano E., Buonfrate D., Pellizzer G., Spolaore P. (2011). Increasing incidence and mortality of infective endocarditis: And population-based study through a record-linkage system. BMC Infect. Dis..

[34] Selton-Suty C., Célard M., Le Moing V., Doco-Lecompte T., Chirouze C., Iung B., Strady C., Revest M., Vandenesch F., Bouvet A. (2012). Preeminence of Staphylococcus aureus in Infective Endocarditis: A 1-Year Population-Based Survey. Clin. Infect. Dis..

[35] Sy R.W., Kritharides L. (2010). Health care exposure and age in infective endocarditis: Results of a contemporary population-based profile of 1536 patients in Australia. Eur. Heart J..

[36] Athan E., Chu V.H., Tattevin P., Selton-Suty C., Jones P., Naber C., Miró J., Ninot S., Fernández-Hidalgo N., Durante-Mangoni E. (2012). Clinical Characteristics and Outcome of Infective Endocarditis Involving Implantable Cardiac Devices. JAMA.

[37] Mills M.T., Al-Mohammad A., Warriner D.R. (2022). Changes and advances in the field of infective endocarditis. Br. J. Hosp. Med..

[38] Wurcel A.G., Anderson J.E., Chui K.K.H., Skinner S., Knox T.A., Snydman D.R., Stopka T.J. (2016). Increasing Infectious Endocarditis Admissions Among Young People Who Inject Drugs. Open Forum Infect. Dis..

[39] Shah A.S.V., McAllister D.A., Gallacher P., Astengo F., Rodríguez Pérez J.A., Hall J., Lee K.K., Bing R., Anand A., Nathwani D. (2020). Incidence, Microbiology, and Outcomes in Patients Hospitalized With Infective Endocarditis. Circulation.

[40] Miao H., Zhang Y., Zhang Y., Zhang J. (2024). Update on the epidemiology, diagnosis, and management of infective endocarditis: A review. Trends Cardiovasc. Med..

[41] Fowler V.G., Miro J.M., Hoen B., Cabell C.H., Abrutyn E., Rubinstein E., Corey G.R., Spelman D., Bradley S.F., Barsic B. (2005). *Staphylococcus aureus* endocarditis: A consequence of medical progress. JAMA.

[42] Urina-Jassir M., Jaimes-Reyes M.A., Martinez-Vernaza S., Quiroga-Vergara C., Urina-Triana M. (2022). Clinical, Microbiological, and Imaging Characteristics of Infective Endocarditis in Latin America: A Systematic Review. Int. J. Infect. Dis..

[43] Grapsa J., Blauth C., Chandrashekhar Y.S., Prendergast B., Erb B., Mack M., Fuster V. (2022). *Staphylococcus aureus* Infective Endocarditis: JACC Patient Pathways. JACC Case Rep..

[44] Tong S.Y., Davis J.S., Eichenberger E., Holland T.L., Fowler V.G. (2015). Staphylococcus aureus infections: Epidemiology, pathophysiology, clinical manifestations, and management. Clin. Microbiol. Rev..

[45] Miro J.M., Anguera I., Cabell C.H., Chen A.Y., Stafford J.A., Corey G.R., Olaison L., Eykyn S., Hoen B., Abrutyn E. (2005). Staphylococcus aureus native valve infective endocarditis: Report of 566 episodes from the International Collaboration on Endocarditis Merged Database. Clin. Infect. Dis..

[46] Cosgrove S.E., Sakoulas G., Perencevich E.N., Schwaber M.J., Karchmer A.W., Carmeli Y. (2003). Comparison of mortality associated with methicillin-resistant and methicillin-susceptible Staphylococcus aureus bacteremia: A meta-analysis. Clin. Infect. Dis..

[47] Jensen A.D., Østergaard L., Petersen J.K., Graversen P., Butt J.H., Bundgaard H., Moser C., Smerup M.H., Modrau I.S., Iversen K. (2022). Surgical treatment of patients with infective endocarditis: Changes in temporal use, patient characteristics, and mortality-a nationwide study. BMC Cardiovasc. Disord..

[48] de Sousa C., Ribeiro R.M., Pinto F.J. (2021). The burden of infective endocarditis in Portugal in the last 30 years—A systematic review of observational studies. Rev. Port. Cardiol. Engl. Ed..

[49] Olaison L., Pettersson G. (2002). Current best practices and guidelines indications for surgical intervention in infective endocarditis. Infect. Dis. Clin. N. Am..

[50] Chu V.H., Cabell C.H., Benjamin D.K., Kuniholm E.F., Fowler V.G., Engemann J., Sexton D.J., Corey G.R., Wang A. (2004). Early Predictors of In-Hospital Death in Infective Endocarditis. Circulation.

[51] Dohmen P.M., Binner C., Mende M., Daviewala P., Etz C.D., Borger M.A., Misfeld M., Eifert S., Mohr F.W. (2016). Gender-Based Long-Term Surgical Outcome in Patients with Active Infective Aortic Valve Endocarditis. Med. Sci. Monit..

[52] Habib G., Hoen B., Tornos P., Thuny F., Prendergast B., Vilacosta I., Moreillon P., Antunes M.d.J., Thilen U., Lekakis J. (2009). Guidelines on the prevention, diagnosis, and treatment of infective endocarditis (new version 2009): The Task Force on the Prevention, Diagnosis, and Treatment of Infective Endocarditis of the European Society of Cardiology (ESC). Endorsed by the European Society of Clinical Microbiology and Infectious Diseases (ESCMID) and the International Society of Chemotherapy (ISC) for Infection and Cancer. Eur. Heart J..

[53] Rocha Carvalho P., Bernardo M., Moreira I., Goncalves F., Fontes P., Moreira J.I. (2023). Predictors of in-hospital mortality in infectious endocarditis. Eur. Heart J. Acute Cardiovasc. Care.

[54] Scheggi V., Merilli I., Marcucci R., Del Pace S., Olivotto I., Zoppetti N., Ceschia N., Andrei V., Alterini B., Stefàno P.L. (2021). Predictors of mortality and adverse events in patients with infective endocarditis: A retrospective real world study in a surgical centre. BMC Cardiovasc. Disord..

[55] Tzoumas A., Sagris M., Xenos D., Ntoumaziou A., Kyriakoulis I., Kakargias F., Liaqat W., Nagraj S., Patel R., Korosoglou G. (2025). Epidemiological Profile and Mortality of Infective Endocarditis Over the Past Decade: A Systematic Review and Meta-Analysis of 133 Studies. Am. J. Cardiol..

[56] Armiñanzas C., Fariñas-Alvarez C., Zarauza J., Muñoz P., González Ramallo V., Martínez Sellés M., Meda J.M.M., Pericás J.M., Goenaga M.Á., Burgos G.O. (2019). Role of age and comorbidities in mortality of patients with infective endocarditis. Eur. J. Intern. Med..

[57] Roubez E., Mouhat B. (2026). Hospital stay duration in infective endocarditis: Descriptive analysis, predictive factors and prognostic impact of early discharge. Arch. Cardiovasc. Dis..

[58] Ahtela E., Oksi J., Vahlberg T., Sipilä J., Rautava P., Kytö V. (2021). Short- and long-term outcomes of infective endocarditis admission in adults: A population-based registry study in Finland. PLoS ONE.

[59] Bishara J., Leibovici L., Israel D.G., Sagie A., Kazakov A., Miroshnik E., Ashkenazi S., Pitlik S. (2001). Long-Term Outcome of Infective Endocarditis: The Impact of Early Surgical Intervention. Clin. Infect. Dis..

[60] Morita Y., Haruna T., Haruna Y., Nakane E., Yamaji Y., Hayashi H., Hanyu M., Inoko M. (2019). Thirty-Day Readmission After Infective Endocarditis: Analysis From a Nationwide Readmission Database. J. Am. Heart Assoc..

[61] Badran A., Rowe H., Jaffar-Karballai M., Abdelghaffar M., Harky A., Yam T.S., Ohri S.K. (2024). A Single-Centre Experience of the Management of Infective Endocarditis. Heart Lung Circ..

[62] Zaman J., Amritphale A., Malozzi C., Amritphale N., Sehgal M., Bassam O. (2021). Costs and predictors of early readmissions in patients with Infective Endocarditis. Utilizing the Nationwide Readmission Database. medRxiv.

[63] Heredia-Rodríguez M., Hernández A., Bustamante-Munguira J., Álvarez F.J., Eiros J.M., Castrodeza J., Tamayo E. (2018). Evolution of the Incidence, Mortality, and Cost of Infective Endocarditis in Spain Between 1997 and 2014. J. Gen. Intern Med..

[64] Sunder S., Grammatico-Guillon L., Baron S., Gaborit C., Bernard-Brunet A., Garot D., Legras A., Prazuck T., Dibon O., Boulain T. (2015). Clinical and economic outcomes of infective endocarditis. Infect. Dis..

