# Supplementary material for: Infective Endocarditis in Ireland: A Nationwide Analysis
Source: J Clin Med. 2026 Aug 24;15(17):6525. doi: 10.3390/jcm15176525 (PMC13566801; doi:10.3390/jcm15176525)
Supplement: Supplementary file 1 [file jcm-15-06525-s001.zip › jcm-4412369-supplementary.pdf]

Supplementary material

Supplementary Table S1: Characteristics of participants by surgical intervention and multivariable logistic regression analysis - Sensitivity analysis excluding length of stay >100 days

		**Adjusted OR (95% CI)**	**P-value**
**Gender**	Female	Reference	
	Male	1.48 (0.79–2.80)	0.222
**Age in years**		0.96 (0.95–0.98)	<0.001
**Charlson Comorbidity Index**		1.02 (0.99–1.05)	0.077

Supplementary Table S2: Characteristics of participants by inpatient mortality and multivariable logistic regression analysis - Sensitivity analysis excluding length of stay >100 days

		**Adjusted OR (95% CI)**	**P-value**
**Gender**	Female	Reference	0.773
	Male	1.12 (0.50-2.51)	
**Age in years**		1.01 (0.98-1.03)	0.537
**Charlson Comorbidity Index**		1.10 (1.06-1.13)	<0.001
**Surgery**		0.97 (0.38-2.46)	0.951
